# Children’s success at detecting circular explanations and their interest in future learning

**DOI:** 10.3758/s13423-016-1195-2

**Published:** 2017-02-07

**Authors:** Candice M. Mills, Judith H. Danovitch, Sydney P. Rowles, Ian L. Campbell

**Affiliations:** 1 School of Behavioral and Brain Sciences, The University of Texas at Dallas, 800 West Campbell Road, GR41, Richardson, TX 75080-3021 USA; 2 Department of Psychological and Brain Sciences, University of Louisville, Louisville, KY 40292 USA; 3 School of Education, Boston University, Boston, MA 02215 USA

**Keywords:** Cognitive development, Knowledge, Circularity, Explanation, Information-seeking

## Abstract

These studies explore elementary-school-aged children’s ability to evaluate circular explanations and whether they respond to receiving weak explanations by expressing interest in additional learning. In the first study, 6-, 8-, and 10-year-olds (*n* = 53) heard *why* questions about unfamiliar animals. For each question, they rated the quality of single explanations and later selected the best explanation between pairs of circular and noncircular explanations. When judging single explanations, 8- and 10-year-olds, and to some extent 6-year-olds, provided higher ratings for noncircular explanations compared to circular ones. When selecting between pairs of explanations, all age groups preferred noncircular explanations to circular ones, but older children did so more consistently than 6-year-olds. Children who recognized the weakness of the single circular explanations were more interested in receiving additional information about the question topics. In Study 2, all three age groups (*n* = 87) provided higher ratings for noncircular explanations compared to circular ones when listening to responses to *how* questions, but older children showed a greater distinction in their ratings than 6-year-olds. Moreover, the link between recognizing circular explanations as weak and interest in future learning could not be accounted for solely by individual differences in verbal intelligence. These findings illustrate the developmental trajectory of explanation evaluation and support that recognition of weak explanations is linked to interest in future learning across the elementary years. Implications for education are discussed.

Explanations are essential for developing an understanding of the world (e.g., Wellman, 2011; see also Keil, 2011; Lombrozo, 2011). In addition to facilitating causal reasoning and understanding (e.g., Amsterlaw & Wellman, 2006), the act of generating explanations guides discovery and constrains inferences in both adults (Fukaya, 2013; Williams & Lombrozo, 2013) and children (see Legare, 2014; Walker, Williams, Lombrozo, & Gopnik, 2012). However, children may not always be willing or able to generate their own explanations. Thus, the ability to evaluate the quality of the explanations they receive from others is essential to learning and decision making.

Past research supports that elementary school-aged children are sensitive to some aspects of the quality of the explanations they encounter. Most of this research has presented children with two explanations pitted directly against each other, finding that children prefer high-quality explanations to extremely low-quality ones. By age 4, children can detect which of two informants made a logically inconsistent statement (e.g., a box is full and empty at the same time), and by age 5, they show greater trust in an informant whose statements are logically consistent (Doebel, Rowell, & Koenig, 2016). By age 7, children prefer logically consistent explanations for physical events to logically inconsistent ones (Samarapungavan, 1992), and they prefer noncircular explanations (i.e., explanations that provide meaningful new information, such as “arctic hares have white fur because it helps them hide in the snow”) to circular ones (i.e., explanations that reiterate the information in the original question without adding any meaningful new information, such as “arctic hares have white fur because their fur is white”; Baum, Danovitch, & Keil, 2008). In some circumstances, even preschool-aged children prefer noncircular explanations to circular ones, such as when the explanations involve familiar concepts (e.g., why it rains; Corriveau & Kurkul, 2014) or simple arguments regarding episodic knowledge (e.g., where a dog is located; Castelain, Bernard, Van der Henst, & Mercier, 2015; Mercier, Bernard, & Clément, 2014).

Although asking children to choose which of two explanations is best or most helpful provides some sense of whether children can discriminate between explanations of varying relative quality, this method does not address whether children can recognize a weak explanation without having a direct comparison to a stronger explanation. In everyday life, children are rarely presented with multiple answers to their queries at the same time. Instead, parents and teachers present one explanation, and children must decide whether the explanation is adequate. Our perspective—and one that is central to the studies presented here—is that a crucial part of learning is recognizing that an explanation is incomplete or uninformative. Indeed, according to some researchers, encountering an information gap leads to a feeling of deprivation (Jirout & Klahr, 2012; Loewenstein, 1994). The feeling can be resolved either by attempting to engage in additional learning to fill the gap or by forgoing future learning because the gap seems too large to resolve (see also Keil, 2006). There is a popular belief that recognizing the limitations of the information you have spurs scientific exploration and learning; however, this question has undergone limited empirical investigation.

Therefore, the studies presented here focused on two issues: how recognizing a weak explanation presented in isolation changes across development, and how recognition of a weak explanation relates to interest in further learning. As noted earlier, very little research has examined children’s ability to recognize weak explanations presented individually. Past research has found that by age 6 or 7, children can detect logical inconsistencies in very simple individual claims (e.g., that a glass cannot be full and empty at the same time; Morris & Hasson, 2010; Ruffman, 1999). There is also evidence that preschool-aged children appear less satisfied when their questions are addressed with nonexplanatory responses that are not actually answers (e.g., restating the question, providing a personal reaction) than with real explanations (Frazier, Gelman, & Wellman, 2009), and that they show better recall for causal explanations (Frazier, Gelman, & Wellman, in press). In these lines of research, however, the claims are either very clearly incorrect or very clearly nonexplanations, which should be much easier for children to evaluate than a weak, yet on-topic, explanation presented in isolation. To better understand the developmental trajectory of children’s ability to recognize circular explanations as less informative answers to causal questions than noncircular explanations, we conducted two studies examining children’s ability to evaluate single explanations.

Presenting children with single explanations is necessary to gather evidence related to our second goal: identifying whether children who recognize that an explanation is weak show greater interest in further learning. The few studies that have examined whether children seek out new information after receiving poor explanations have involved either nonexplanations for unusual behaviors (Frazier et al., 2009) or simple mechanical explanations (Legare, 2012). Although these studies demonstrate that weak responses to questions prompt exploration, even the best responses that children receive in these studies are still very simple, and thus “gaps” are easy to detect. In reality, scientific explanations are typically iterative, so there are always greater levels of detail to be explored (e.g., Keil, 2006). To address this issue, our studies used explanations with a greater degree of potential depth and detail: explanations regarding complex biological phenomena. After evaluating a series of explanations, children had the opportunity to take home additional information about those topics, allowing us to examine the link between explanation evaluation and interest in further learning. We expected that children’s ability to recognize that circular explanations contained gaps would relate to their interest in learning more in the future.

## Study 1

In this study, children heard questions and then evaluated circular or noncircular explanations provided for those questions, both individually and in contrasting pairs. To avoid influencing children’s sensitivity to single weak explanations, children judged all the single explanations before choosing between the contrasting pairs. We also examined the relation between children’s ability to recognize circular explanations and their interest in seeking out additional information about the question topics.

### Method

#### Participants

Participants were twenty 6-year-olds (*M*_age_ = 6.04 years, *SD* = .67; 10 females), seventeen 8-year-olds (*M*_age_ = 8.06 years, *SD* = .54; nine females), and sixteen 10-year-olds (*M*_age_ = 10.01 years, *SD* = .52; eight females) from the Dallas area. Demographically, 76 % of the participants identified as Caucasian, 13 % as Asian, 10 % as Black or African American, and less than 1 % as other races.

#### Design

The stimuli were questions about eight animals likely to be unfamiliar to children (e.g., pangolin, colugo). Two *why* questions were developed for each animal, each with a related circular and noncircular explanation, for a total of 16 questions (see Appendix). Circular explanations reiterated information from the original question without adding any meaningful new information, whereas noncircular explanations provided meaningful new information. For instance, children heard someone ask, “Why do pangolins climb trees?” The circular explanation was: “Pangolins climb trees because there are trees around for them to climb.” The noncircular explanation was: “Pangolins climb trees because they eat insects that live in trees.” Mirroring previous research (e.g., Corriveau & Kurkul, 2014), circular and noncircular explanations were matched for length and complexity, as measured by Flesch Reading Ease scores (Flesch, 1948) (*M*_circular_ = 76.1, *M*_noncircular_ = 73.6), *t*(15) = 1.66, *p* = .12. These questions were randomly divided into two sets, with each set containing one question per animal. The order of the question sets was counterbalanced across participants, and each set was used an equal number of times in each phase. Each experimental session consisted of three phases: single explanation, paired explanation, and information seeking. Note that for the single explanation phase, children heard eight different questions with different explanations (i.e., they never heard a circular and noncircular explanation for the same question). Presentation software was used to present images of the animals and audio recordings (standardized for length) of a female reading the related explanations for both explanation phases.

#### Materials and procedure

Participants were tested individually. Each child was told that “Jane” saw pictures of strange but real animals, had questions about the animals, and asked multiple people for answers to her questions. The child was then told that of the people Jane asked, “some people seemed to know a lot, some only knew a little bit, and some didn’t seem to know very much at all.” The experimenter explained that Jane wanted the child to decide how well each explanation she heard answered her questions.

#### Single explanation phase

Children were introduced to a 5-point Likert scale for indicating the quality of each explanation. The scale consisted of pictures ranging from two thumbs pointing up to two thumbs pointing down, with a sideways thumb as the midpoint. Children were told that two thumbs up represented an explanation that answered the question *really well and really helps you understand something*, two thumbs down represented an explanation that *does not answer the question at all and does not help you understand something*, and a sideways thumb represented an explanation that *answers the question somewhere in the middle—it gives some helpful information but not all the way.*

Following the instructions, the experimenter presented and labeled a picture of an animal on the computer screen and stated Jane’s question. Then a stick figure appeared, representing someone Jane had asked. The experimenter introduced this person (i.e., “Someone said . . ”) and played the audio recording of the response while a speech bubble appeared next to the stick figure. Children then indicated how well the explanation answered Jane’s question using the thumb scale.

At the beginning of this phase, children completed two practice questions to familiarize them with the question and explanation process. The practice questions and explanations did not overlap with the test items. Practice questions were the same for every child, with the first question receiving a noncircular explanation and the second question receiving a circular explanation. The goal of the practice questions was to sensitize children to the fact that the explanations could vary in quality without training them on how to respond. Children did not receive feedback on their ratings for the practice questions, and these ratings were excluded from analysis.

The test items were then presented, followed by two check items featuring familiar animals that were intended to verify whether children could use the scale appropriately. The first check item involved an irrelevant explanation where the causal mechanism was unclear and the second involved a solid causal explanation (see Appendix).

#### Paired explanation phase

Children were told that they would now hear two different explanations in response to Jane’s question. Children were instructed to choose which explanation answered the question best, and to indicate how much better that explanation was than the other one (i.e., a lot better or a little better). For each question, children were presented with one noncircular explanation and one circular explanation, which were counterbalanced for order across conditions. The procedure was otherwise similar to Phase 1, except that two stick figures were presented on the screen at once, with a speech bubble appearing for each figure when that figure provided an explanation.

#### Information-seeking phase

Eight animal cards (corresponding to each target animal) with a picture of the animal on one side and facts about the animal on the other side were placed on the table. Children were told that the cards had information about the animals that they had talked about, that there were plenty of copies, and that they could keep as many or as few cards as they wanted. The number of cards each child took was recorded.

### Results and discussion

#### Single explanation phase

Children’s ratings on the thumb scale were calculated with two thumbs down assigned a value of 1 and two thumbs up assigned a value of 5. A 2(explanation type: circular vs. noncircular) × 3(age group: 6-year-olds, 8-year-olds, 10-year-olds) mixed-measures ANOVA was conducted on the average ratings.

**Fig. 1:**
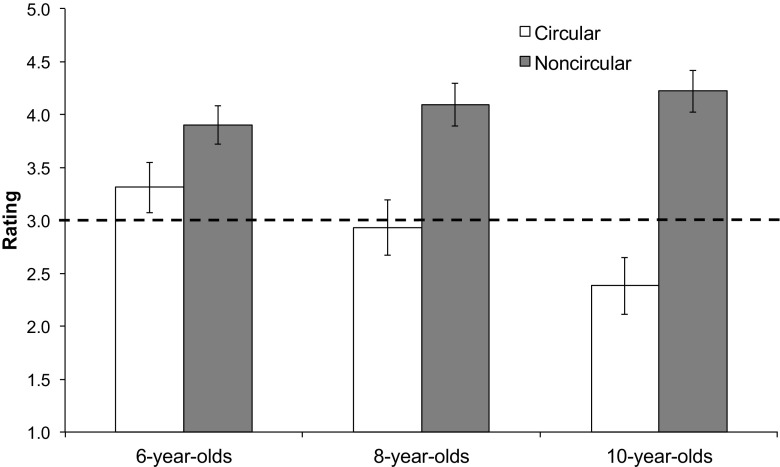
Average ratings for single explanations in Study 1, with higher ratings indicating higher quality explanations. The *dashed line* indicates the midpoint of the scale

We found a main effect of explanation type, with children rating noncircular explanations as higher in quality than circular explanations, *F*(1, 50) = 48.92, *p* < .001, η_p_^2^ = .50. We also found an explanation type by age group interaction, *F*(2, 50) = 4.56, *p* = .02, η_p_^2^ = .15, with older children showing a greater distinction in the ratings than the 6-year-olds (see Fig. 1).

Post hoc paired *t* tests comparing average ratings for circular and noncircular explanations for each age group revealed that 8- and 10-year-olds rated noncircular explanations as significantly better than circular explanations, *t*s > 4.03, *p*s < .001. (All *t* tests reported here and throughout the article were two-tailed tests.) However, 6-year-olds did not do so at statistically significant levels, *t*(19) = 1.76, *p* = .09. That said, we note that some might consider this a statistical trend, and inspection of individual patterns of data below supports that *some* of the 6-year-olds rated noncircular explanations as better than circular ones.

In addition, independent-samples *t* tests were used to determine if children in each age group rated the circular explanations as being significantly lower and the noncircular explanations as being significantly higher than the midpoint of the scale. All age groups rated the noncircular explanations significantly higher than the midpoint, *t*s > 4.34, *p*s < .001, suggesting that they viewed them as helpful. In contrast, only the 10-year-olds rated the circular explanations as significantly lower than the midpoint of the scale, *t*(15) = 2.16, *p* = .048, Cohen’s *d* = 1.12; the 6-year-olds and 8-year-olds did not, *p*s > .23.

Because the check items were added after several participants had been tested, we analyzed the check explanation ratings for 41 children. Eight-year-olds and 10-year-olds gave a lower rating to the irrelevant check explanation than to the solid check explanation, *t*s > 4.45, *p*s < .005, supporting that they used the scale as anticipated during the study. Performance was less clear for the 6-year-olds, who did not show a significant difference in ratings for the two types of items as a group, *t*(14) = .91, *p* = .38. Of the fifteen 6-year-olds who completed the posttest items, eight gave the irrelevant explanation a lower rating than the solid explanation, but there was a great deal of variability in performance for these items. As expected, all age groups rated the solid check explanation higher than the midpoint of the scale, *t*s > 3.67, *p*s < .005. The older age groups rated the weak explanation lower than the midpoint of the scale, *t*s > 4.45, *p*s < .005. The 6-year-olds did not, although they trended in that direction, *t*(14) = 1.85, *p* = .09.

#### Paired explanation phase

**Fig. 2:**
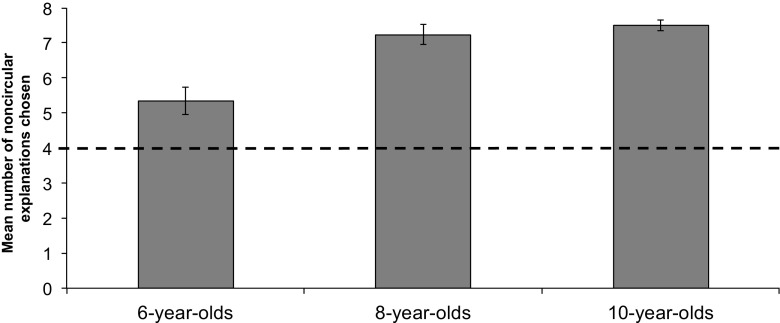
Mean number of times a noncircular explanation was chosen over a circular explanation in the paired explanation phase in Study 1. The *dashed line* indicates chance performance

Examining the number of times children of each age group identified the noncircular explanation as more helpful than the circular explanation (out of eight trials), a one-way ANOVA with age group as a between-subjects factor revealed a significant difference between the age groups, *F*(2, 50) = 15.27, *p* < .001, η_p_^2^ = .38. Bonferroni-corrected post hoc tests found that 6-year-olds selected significantly fewer noncircular explanations than the 8- and 10-year-olds, *p*s < .001. However, one-sample *t* tests (chance = 4) indicated that all age groups selected the noncircular explanations over the circular explanations at rates that exceeded chance, *t*s > 3.49, *p*s < .003 ( see Fig. 2).

The strength of children’s preference for each noncircular explanation was analyzed by converting responses to a 4-point scale (e.g., 4 indicated preferring the noncircular explanation *a lot*). Findings mirrored the previous analysis: 6-year-olds showed a weaker preference for noncircular over circular explanations than the 8- and 10-year-olds, *F*(2, 52) = 9.85, *p* < .001, η_p_^2^= .28, but all age groups preferred the noncircular explanations at rates greater than chance (chance = 2.5), *t*s > 3.46, *p*s < .004.

#### Comparison between single and paired explanation phases

We compared ratings for noncircular explanations across the single and paired explanation phases in two different ways. First, we correlated the difference between each child’s mean noncircular and circular ratings in Phase 1 with the child’s overall preference for noncircular explanations in Phase 2, finding a significant relation, *r* = .42, *p* = .002. Thus, children who made a greater distinction between the circular and noncircular single explanations in Phase 1 were also more likely to prefer the noncircular explanations relative to circular explanations in Phase 2.

Second, we calculated the number of children in each age group who successfully distinguished between the explanations for each phase (see Table 1). For the single explanation phase, successful performance consisted of rating the noncircular explanations higher than the circular ones, on average. For the paired explanation phase, successful performance consisted of preferring noncircular explanations more often than circular ones. We anticipated that children would perform better in the paired explanation phase than the single explanation phase, particularly the younger children (who we expected might struggle more to recognize a weak explanation when it is presented on its own as opposed to in contrast with another). Notably, the two older age groups tended to show consistent successful performance on both tasks. In contrast, the 6-year-olds were less consistent. In general, they were more likely to be successful for paired explanations than for single explanations.

**Table 1:** Percentage of children who fell into specific category of performance for single and paired explanation phases

	Successful in both phases	Successful in single only	Successful in paired only	Successful in neither phase
6-year-olds	40	15	30	15
8-year-olds	94	0	6	0
10-year-olds	100	0	0	0

#### Information seeking

Finally, we examined patterns of interest in animal information cards for children who completed the card selection task (*n* = 45). A one-way ANOVA revealed no age differences in the number of cards taken, *F*(2, 44) = .84, *p* = .44. Children took an average of five cards, but there was significant variability (*M* = 5.02, *SD* = 2.55). Of the children who completed the information-seeking task, there was a nonsignificant relationship between the ratings of the circular explanations and the number of cards they took, *r*(45) = -.22, *p* = .16.

Because of evidence that 6-year-olds did not regularly distinguish between weak and strong explanations for the study questions as well as for the check items, further analyses focused on the older children only (*n* = 30). We found that the lower children rated the circular explanations, the more cards they took, *r*(29) = -.43, *p* = .02. In other words, children who recognized the weakness of the circular explanations wanted to acquire more information about the animals than children who did not recognize the weakness of the circular explanations. Notably, children did not only select the animals for which they had received circular explanations; indeed, some children took all eight cards. We found no relation between the number of cards taken and the rating of noncircular explanations in the single explanation phase or the preference for the noncircular explanation in the paired explanation phase (*r*s < .24, *p*s > .19). This may be in part because there was less variability in those ratings, with the majority of children rating the noncircular explanations as high in quality.

Taken together, these findings demonstrate that for single explanations, the 8- and 10-year-old children (and, in some cases, 6-year-old children) clearly distinguished between noncircular and circular explanations in their ratings, and only the 10-year-olds rated the circular explanations as being poor in quality (as opposed to neutral or strong). For paired explanations, all three age groups successfully identified noncircular explanations as more helpful than circular ones. However, the middle and oldest age groups performed near ceiling, identifying the most informative explanation in a pair more frequently than the youngest children. In other words, in both phases, 6-year-olds found it more difficult than older children to recognize that circular explanations were weak.

## Study 2

Study 1 provided evidence that identifying weak explanations is more challenging for children when they do not have a stronger explanation for comparison. To further examine children’s ability to evaluate single explanations, Study 2 used a different rating scale to emphasize the informative nature of explanations and included a larger number and variety of check items to determine whether children use the scale to rate explanations appropriately.

A possible explanation for the challenges children faced in rating single explanations (particularly for 6-year-olds) in Study 1 is that questions starting with *why* are often difficult to briefly and meaningfully answer. For example, adults may answer a question about why pangolins have scales by explaining the scales’ protective function, but they could also explain how the mechanism evolved, or that a higher power made it so (see Evans, 2001). The variability in explanatory responses to *why* questions that children typically encounter may make it more challenging for children to detect a weak response. To address this issue, Study 2 focused on *how* questions that can be answered in terms of straightforward causal mechanisms.

An additional issue of interest in Study 2 is the relation between explanation evaluation and future learning observed in Study 1. Study 2 aimed to replicate and extend this finding using the information-seeking measure from Study 1 as well as an item-based self-report interest rating measure. Study 2 also explored a third variable that may explain this link. Perhaps children who have larger vocabularies and more semantic knowledge are better at recognizing explanatory gaps and are also more motivated to learn. In other words, perhaps the link between recognizing weak explanations and interest in learning more information is due to verbal intelligence instead of the ability to recognize a weak explanation. This study examined this possibility.

### Method

#### Participants

Participants were thirty-one 6-year-olds (*M*_age_ = 5.84 years, *SD* = .59; 15 females), twenty-seven 8-year-olds (*M*_age_ = 7.99 years, *SD* = .61; 12 females), and 29 10-year-olds (*M*_age_ = 9.60 years, *SD* = .40; 17 females) from Dallas, Texas, and Louisville, Kentucky. Demographically, 63 % of the participants identified as Caucasian, 13 % as Asian, 7 % as Black or African American, and 7 % as other races (10 % did not report this information). Two additional participants were tested but excluded from the analysis (one for not completing the study, one for scoring lower than three standard deviations below the mean on the intelligence measure). Participants from both locations were tested with the same procedures and stimuli, and they did not differ on verbal intelligence scores, *t*(84) = 1.058, *p* = .29.

#### Design

The stimuli consisted of questions about 12 unfamiliar animals (e.g., mudskipper, colugo). Each question was presented with either a circular or noncircular explanation (see Appendix). There were no significant differences in Flesch Reading Ease scores (Flesch, 1948) between the two types of explanations for each question (*M*_circular_ = 93.14, *M*_noncircular_ = 89.73), *t*(12) = 1.07, *p* = .31.

Each experimental session consisted of three phases: single explanation, information-seeking, and verbal intelligence measurement.

#### Materials and procedure

Participants were tested individually. Children heard an introduction identical to that in Study 1.

#### Single explanation phase

Children were presented with a sheet of paper with five identical circles with different amounts of green ink inside them ranging from nearly “empty” to nearly “full” with a midpoint of a half-filled circle. Children were instructed to use the circles to show how well an explanation answered a question, with the empty circle corresponding to explanations that did not give enough information and the full circle corresponding to explanations that gave all necessary information. Children were specifically instructed not to judge whether the explanation was right or true, but whether it did a good job of answering the question. Children’s understanding of the scale was verified by asking them to point to circles corresponding to informative, uninformative, and partially informative explanations.

Children then completed 12 test items. This procedure was identical to the single explanation phase in Study 1, with the addition of an interest question presented after each rating, asking whether the children would want to learn more about that animal, and if so, how much more (e.g., a little or a lot). Following the test items, children completed six check items: two solid, two bizarre, and two nonexplanation (see Appendix). The solid items provided clear causal explanations involving familiar animal characteristics; these were to check if children recognize clearly good quality explanations because they should assign these explanations high ratings. The two weak check items examined different aspects of children’s understanding of weak explanations. The bizarre check items provided explanations that addressed the question but were nonsensical, making them theoretically easy to rate as poor quality. The irrelevant check items provided explanations that were accurate claims about the animal, but not actually relevant to the questions being asked. If children were focused on how clearly the explanations addressed the questions, then they should give these explanations poor ratings, even though they were true statements when considered on their own.

#### Information-seeking phase

This phase was identical to Study 1, except with 12 animal cards.

#### Verbal intelligence measure

Children completed the verbal knowledge and riddles subscales of the *Kaufman Brief Intelligence Test, Second Edition* (KBIT-2; Kaufman & Kaufman, 2004), which yields a score that reflects both vocabulary skills and semantic knowledge.^1^

### Results and discussion

#### Ratings

**Fig. 3:**
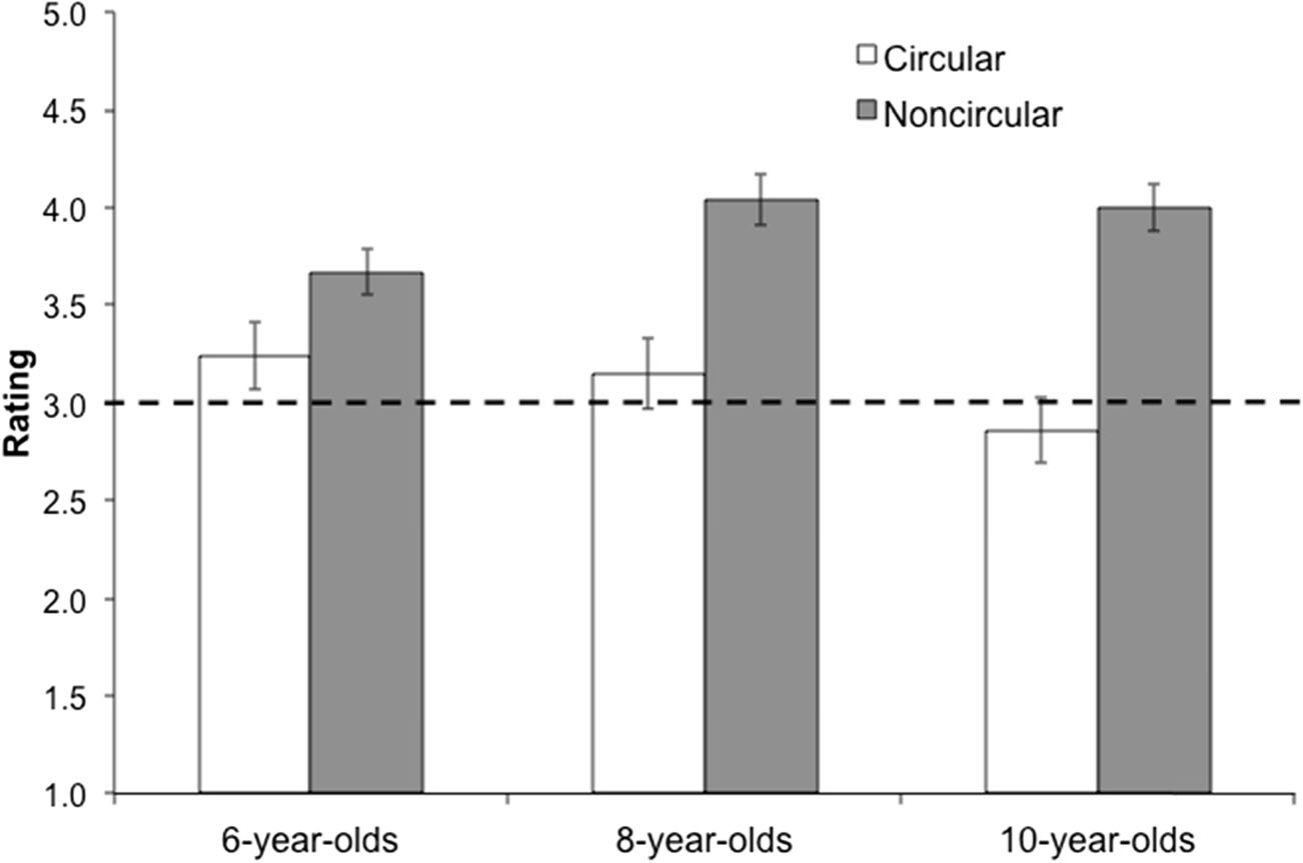
Average ratings for single explanations in Study 2, with higher ratings indicating higher quality explanations

A 2(explanation type: circular vs. noncircular) × 3(age group: 6-year-olds, 8-year-olds, 10-year-olds) mixed-measures ANOVA was conducted on the average ratings. Similar to Study 1, we found a main effect of explanation type, *F*(1, 84) = 59.40, *p* < .001, η_p_^2^ = .41, and an explanation type by age group interaction, *F*(2, 84) = 4.00, *p* = .02, η_p_^2^= .09 (see Fig. 3). Overall, older children showed a greater distinction in their ratings than the 6-year-olds. Post hoc paired *t* tests found that all three age groups rated noncircular explanations as significantly better than circular explanations, *t*s > 2.55, *p*s < .001.

In addition, independent-samples *t* tests compared the average ratings for each explanation type to the midpoint of the scale. No age group rated the circular explanations lower than the midpoint (*t*s < 1.67, *p*s > .11); all age groups rated the noncircular explanations higher than the midpoint (*t*s > 4.81, *p*s < .001). These data suggest that, on average, children did not see the noncircular explanations as particularly weak or empty of information.

#### Check items

Average scores for each type of check item were calculated. Bizarre and irrelevant check items were rated as lower in quality than solid check items for all age groups, *p*s < .001.

As expected, all age groups rated the solid check explanation higher than the midpoint of the scale, *t*s > 5.34, *p*s < .001. Collapsing across the two types of weak check explanations, we found that all age groups rated the weak check explanations as lower than the midpoint of the scale, *t*s > 2.72, *p*s < .02. This confirms that all age groups understood the full range of the scale despite the fact that no age group rated the circular explanations lower than the scale midpoint. In addition, it also worthwhile to note that children provided lower ratings to both types of weak check explanations than to the circular explanations, *t*s > 5.94, *p*s < .001.

### Information seeking

#### Interest ratings

For each child, we calculated the interest rating for the questions for which they had originally heard circular explanations and the questions for which they had originally heard noncircular explanations. An ANOVA with item explanation type (circular vs. noncircular) and age group as a between-subjects factor revealed no significant main effects or interactions. Overall, children reported being somewhere between *a little* and *a lot* interested in learning more about the animals (*M* = 1.23 out of 2). Many children used the same response for all items, suggesting that the interest scale was not sufficiently sensitive.^2^

#### Cards

As in Study 1, a one-way ANOVA revealed no age differences in the number of cards taken, *F*(2, 84) = 1.26, *p* = .29. Children took an average of six cards (of 12), but as with Study 1, there was significant variability (*M* = 6.46, *SD* = 3.70). Also as in Study 1, the lower children rated the circular explanations, the more cards they took, *r*(87) = -.24, *p* = .02; this was also true for both card types separately: the lower children rated the circular explanations, the more circular cards they took (*r* = -.21, *p* = .05) and the more noncircular cards they took (*r* = -.26, *p* = .02).^3^

Eighty-five participants completed the verbal intelligence measure. The average score was around the standardized population mean (*M* = 104.55, *SD* = 12.45), with no age differences, *F*(1, 83) = 1.11, *p* = .34. Notably, scores on the KBIT-2 correlated with the number of cards taken, where children who scored higher on the KBIT-2 took home more cards, *r*(86) = .21, *p* = .049 (see Table 2). Still, even after controlling for KBIT-2 scores, the relationship between the circular ratings and the number of cards taken remained significant, *r*(83) = -.21, *p* = .049.

**Table 2:** Correlation matrix for Study 2

	KBIT-2	Noncircular	Circular	Cards taken
Age	-.164	.251*	-.158	-.163
KBIT-2 standardized score		.069	-.197	.213*
Noncircular rating mean			.212*	-.081
Circular rating mean				-.243*

*p < .05.

## General discussion

Children are often faced with incomplete or inadequate explanations. The studies presented here focused on two primary issues: how recognizing a weak single explanation changes across development, and how recognition of a weak explanation relates to interest in future learning. Related to our first goal, results indicated that although 8- and 10-year-olds were generally successful at rating single circular explanations as weaker than single noncircular ones, 6-year-olds sometimes struggled to do so. In both studies, we observed developmental improvements in rating circular explanations as weaker than noncircular ones.

In characterizing how children evaluated the circular explanations, it is important to note that children did not typically judge circular explanations to be weak. In both studies, each age group rated the circular explanations as being no weaker than the midpoint of the scale—the one exception was the 10-year-olds in Study 1. These judgments do not reflect an unwillingness to use the full range of the scale, as children generally used the low end of the scale in their evaluations of the weak check explanations in both studies. We also believe that these judgments are not due to misinterpreting the scale as measuring something like *accuracy*. If children were using the scale to indicate judgments of accuracy, then they would have rated the irrelevant yet accurate check explanations in Study 2 as moderate to strong. But they did not, appearing to recognize that although those statements were true, they did not appropriately address the questions. In addition, anecdotally, some children also spontaneously referenced the purpose of the scale after hearing circular explanations, making comments like “that doesn’t answer the question!” Thus, the data support that children used the scale to indicate how well each explanation answered the question.

Despite this understanding, children still did not give the circular explanations low ratings. Indeed, although children in these studies recognized that the circular explanations were not as informative as noncircular explanations, most children still rated these empty, unhelpful circular explanations as *passable* (i.e., giving some helpful information or being somewhat informative). Why are children less discriminating toward circular explanations than other kinds of clearly weak explanations? Our speculation is that when children hear a single circular explanation in response to a question, they sometimes struggle to recognize that the explanation does not provide additional information. Particularly for novel questions, it may take significant attention and working memory capacity to keep in mind what the question asked, which may leave fewer mental resources for reflecting on the quality of the explanation.

Given these findings, future research should examine the conditions under which young children recognize that weak explanations are unhelpful. Children’s performance may depend on many factors. For instance, recent findings suggest that the development of executive function and working memory skills contributes to younger children’s ability to evaluate information (Doebel et al., 2016). In our studies, exploratory analyses revealed that sheer amount of knowledge (measured as the raw scores on the KBIT-2) related to children’s ratings of circular explanations, *r*(83) = .237, *p* = .029, after controlling for age. Additionally, in our studies, 6-year-olds were better at rating circular explanations as weaker than noncircular explanations in Study 2 than in Study 1. Although this might be partially due to changes in the scale, we believe it is primarily due to changes in the type of questions (*how* questions in Study 2, *why* questions in Study 1). We speculate that circular explanations and nonexplanations may be more frequent or more acceptable for certain types of questions, such as when explaining conventional behaviors (e.g., why pajamas are worn to bed and not to school). More broadly, there are many different ways that explanations can provide unsatisfactory responses to questions. It will be important for future research to examine how children respond to different kinds of weaknesses in explanations to better understand what is changing across development.

The second goal of these studies related to children’s interest in future learning. In both experiments, the lower children rated the single circular explanations (i.e., the more they recognized that the circular explanations provided little information), the more information cards they took. We propose that when children explicitly recognized that they had been presented with weak explanations that were clearly missing information, they were motivated to seek out more complete information through taking home information cards. Moreover, our findings suggest that this is not solely a function of verbal intelligence.

From these studies alone, it is difficult to determine the specificity of the link between recognition of weak explanations and an interest in additional learning. One possibility is that after encountering a weak explanation to a question, children’s interest in learning is *specific*, such that they will seek additional information that would better answer that particular question. Another possibility is that recognizing weak explanations leads children to have a *general* increased interest in learning; perhaps encountering and recognizing weak explanations leads to a general feeling of information deprivation within the child that can be quenched by encountering complete explanations of any sort. At this point, our findings support the general link, as children were just as interested in gathering additional information about items that had been answered with circular explanations as those that had been answered with noncircular explanations (see Study 2 results). That said, it is important to note that our measures of interest in additional learning involved opportunities for children to acquire general information about each animal rather than answers to specific questions, so it is too early to say whether the specific link is also present.

To achieve educational success, children (and adults) need to be willing to explore the answers to questions in great detail. If an interest in future learning sometimes depends directly on recognition that there are gaps in available information, it would be useful to better understand how to help children recognize that those gaps exist. We speculate that when children recognize that explanations are missing information and/or have gaps (e.g., Mills & Keil, 2004), as long as the conditions are appropriate (e.g., information is available, cognitive load is low), they will purposefully seek out additional information to fill those particular gaps (i.e., there is a specific link between recognizing an explanatory gap and interest in learning) and perhaps related ones (i.e., a more general link). Future research is necessary to better understand how reflection on explanatory quality relates to interest in learning.

In sum, school-aged children recognize that noncircular explanations are better than circular explanations, but younger children have more difficulty recognizing the weakness of circular explanations, particularly when they are presented individually. Given that children typically receive single explanations, these findings suggest that past research may have overestimated young children’s ability to recognize weak explanations. That said, children *can* sometimes recognize that circular explanations are weak, and their ability to recognize weak explanations relates to their information-seeking behaviors. In moving forward, these findings strongly suggest that helping children learn to better recognize weak explanations may serve multiple purposes, both giving them insight into what they do not know and encouraging them to more actively participate in their own education.

## Appendix

### Study 1. Questions and explanations

**Table:**

**Animal**	**Question**	**Explanation**
Pangolin	Why do pangolins climb trees?	**Circular:** Pangolins climb trees because there are trees around for them to climb.
		**Noncircular:** Pangolins climb trees because they eat insects that live in trees.
	Why are a pangolin’s scales hard?	**Circular:** The pangolin has hard scales because when pangolins grow scales, they are hard.
		**Noncircular:** The pangolin has hard scales because they help shield it from animal attacks.
Tapir	Why do tapirs have blurred eyesight?	**Circular:** Tapirs have blurred eyesight because everything they see is blurry to them when they look at things.
		**Noncircular:** Tapirs have blurred eyesight because they have an extra, cloudy layer over their eyes that is hard to see through.
	Why do baby tapirs have spotted coats?	**Circular:** Baby tapirs have spotted coats because they are born with more spots on their coats than adults.
		**Noncircular:** Baby tapirs have spotted coats because the spots help them blend in to the forest and be safe.
Pink fairy armadillo	Why do pink fairy armadillos have thin white hairs on their underbelly?	**Circular:** Pink fairy armadillos have thin white hair on their underbelly because the hair that grows there is thin and white.
		**Noncircular:** Pink fairy armadillos have thin white hair on their underbelly because the hair keeps their body from getting too hot or cold.
	Why do pink fairy armadillos dig underground?	**Circular:** Pink fairy armadillos dig underground because they get on the ground and dig.
		**Noncircular:** Pink fairy armadillos dig underground because they eat ants from there.
Frilled dragon	Why does the frilled dragon run on its back legs?	**Circular:** The frilled dragon runs on its back legs sometimes because it runs on the legs that are on the back side of its body.
		**Noncircular:** The frilled dragon runs on its back legs sometimes because it wants to be tall to scare its enemies as it runs away.
	Why does the frilled dragon have a sticky tongue?	**Circular:** The frilled dragon has a sticky tongue because its tongue sticks to things.
		**Noncircular:** The frilled dragon has a sticky tongue because the stickiness helps it catch insects for food.
Colugo	Why does the colugo have skin flaps?	**Circular:** The colugo has skin flaps because there are flaps of skin on its body.
		**Noncircular:** The colugo has skin flaps because it needs them to glide around treetops.
	Why does the colugo travel for up to 2 miles each night?	**Circular:** The colugo travels for up to 2 miles each night because there are many miles of forest through which it can travel.
		**Noncircular:** The colugo travels for up to 2 miles each night because its food is spread out and it needs to travel that far to find it.
Echidna	Why does the echidna curl up into a ball?	**Circular:** The echidna curls up into a ball because its body makes that shape when it rolls up.
		**Noncircular:** The echidna curls up into a ball because that shields its soft belly from harm.
	Why does the echidna have a thin nose?	**Circular:** The echidna has a thin nose because its nose is skinny and not fat.
		**Noncircular:** The echidna has a thin nose because it makes it easier to catch bugs.
Tarsier	Why can tarsiers leap really far?	**Circular:** Tarsiers can leap really far because they can jump long distances.
		**Noncircular:** Tarsiers can leap really far because they have long, powerful legs.
	Why do tarsiers have huge eyes?	**Circular:** Tarsiers have huge eyes because they are born with two eyes that are very large on their faces.
		**Noncircular:** Tarsiers have huge eyes because they hunt for food at night and big eyes help them see in the dark.
Dana octopus squid	Why does the dana octopus squid flash light at its prey?	**Circular:** The dana octopus squid flashes light at its prey because two of its tentacles have lights on the ends of them.
		**Noncircular:** The dana octopus squid flashes light at its prey because lights blind the prey to make it easier to catch for food.
	Why does the dana octopus squid live so deep underwater?	**Circular:** The dana octopus squid lives very deep underwater because it lives near the floor of the ocean.
		**Noncircular:** The dana octopus squid lives very deep underwater because that is where it finds most of its food.

### Study 2. Questions and explanations

**Table:**

**Animal**	**Question**	**Explanation**
Pink fairy armadillos	How do pink fairy armadillos use the thin white hair on their underbellies to stay healthy?	**Circular:** Their underbellies have thin white hair that helps them not get sick.
		**Noncircular:** Their thin white hair keeps their bodies from getting too hot or cold.
Colugo	How do colugos use their skin flaps to travel?	**Circular**: Their skin flaps help them to move from one place to another.
		**Noncircular:** Their skin flaps allow them to glide from treetop to treetop.
Tarsier	How do tarsiers use their huge eyes to stay alive?	**Circular:** Their huge eyes can help them to live a lot longer.
		**Noncircular:** Their huge eyes help them find their food in the dark.
Wombat	How do wombats use their backwards pouches to keep their babies clean?	**Circular:** Their backwards pouches help them keep their babies from getting dirty.
		**Noncircular:** Their backwards pouches face their babies away from the dirt wombats dig up.
Thorny dragon	How do thorny dragons use the grooves between their thorns to help them drink water?	**Circular:** Their thorns have grooves that can help them drink water.
		**Noncircular:** Their grooves collect water and send the water to their mouths.
Saiga antelope	How do saiga antelopes use their noses to keep their lungs clean?	**Circular:** Their noses are able to keep their lungs from getting dirty.
		**Noncircular:** Their noses have special filters that stop dirt from reaching their lungs.
Mudskipper	How do mudskippers breathe when there is little air?	**Circular:** They can breathe even when there is not much air around them.
		**Noncircular:** They store a pocket of air inside of them to breathe from.
Gerenuk	How do gerenuks get the water they need without drinking?	**Circular:** They do not drink in order to get the water they need.
		**Noncircular:** They get the water they need by eating plants that have water inside.
Aye-aye	How do aye-ayes use their fingers to find hidden food?	**Circular:** They can use their fingers to know where their food is hidden.
		**Noncircular:** They tap hollow trees with their fingernails to hear bugs inside.
Racket-tailed drongo	How do racket-tailed drongos use their voices to steal food?	**Circular:** They use their voice to help them take food that they can eat.
		**Noncircular:** They copy alarm sounds of animals to scare them and steal their food.
Yeti crab	How do yeti crabs’ hairy arms keep them safe in poisonous water?	**Circular:** Their hairy arms keep them from getting harmed in poisonous water.
		**Noncircular:** Their hairy arms have helpful germs that clean the water around them.

### Study 1. Check items and explanations

**Table:**

**Animal**	**Question**	**Explanation**
Rabbit	Why do rabbits eat carrots?	**Irrelevant:** Rabbits eat carrots because they have two ears.
Dog	Why do dogs need sleep?	**Solid explanation:** Dogs need sleep because their bodies need to rest to gather energy for the next day.

### Study 2. Check items and explanations

**Table:**

**Animal**	**Question**	**Explanation**
Tapir	How do baby tapirs use their spotted coats to hide?	**Solid explanation:** Their spotted coats help them blend into the forest.
Pangolin	How do the pangolin’s scales protect them from predators?	**Solid explanation:** Their scales form a hard shell around their body that is hard to break through.
Okapi	How does the okapi use its stripes as protection?	**Irrelevant:** The okapi has fur that looks like chocolate.
Blue glaucus	How does floating upside down help the blue glaucus survive?	**Irrelevant:** The blue glaucus looks like a snowflake.
Gobi jerboa	How does the Gobi jerboa use its ears to stay cool?	**Bizarre explanation:** The Gobi jerboa uses its ears to buy ice from the supermarket.
Shoebill	How does standing still help the shoebill get food?	**Bizarre explanation:** The shoebill stands still so that it is able to find the nearest restaurant.

## Footnotes

1 An additional measure of motivational framework was administered at the end of the study, but it is beyond the scope of this article.

2 To examine the relationship between interest ratings and ratings of circular explanations for individual items, a multilevel model analysis was conducted, finding no significant relationship. However, this analysis should be considered tentative, as the categorical dependent variable and the sample size constrain this analysis.

3 To examine the relationship between ratings of each circular explanation and whether or not a card was chosen for that explanation, a multilevel model analysis was conducted, finding no significant relationship. However, this analysis should also be considered tentative due to constraints based on the binary dependent variable and the sample size. Exploratory analyses suggest that children may be slightly more likely to choose a card for explanations they rated as weak (ratings of 1 or 2; 59.3 % of the time) than for explanations they rated as acceptable or good (ratings of 3 through 5; 50 % of the time).
